# Efficacy and Safety of an Inactivated Phase I *Coxiella burnetii* Vaccine to Control Q Fever in Ruminants: A Systematic Review

**DOI:** 10.3390/ani14101484

**Published:** 2024-05-16

**Authors:** Philippe Gisbert, Ana Hurtado, Raphaël Guatteo

**Affiliations:** 1 Ceva Santé Animale, 33500 Libourne, France; 2 Animal Health Department, NEIKER—Basque Institute for Agricultural Research and Development, Basque Research and Technology Alliance (BRTA), Bizkaia Science and Technology Park 812L, 48160 Derio, Spain; ahurtado@neiker.eus; 3 Oniris, INRAE, BIOEPAR, 44300 Nantes, France; raphael.guatteo@oniris-nantes.fr

**Keywords:** *Coxiella burnetii*, Q fever, vaccine, vaccination, ruminants, cattle, goats, sheep

## Abstract

**Simple Summary:**

Simple Summary: Q fever is a disease that affects many animal species, including humans. It is caused by the intracellular bacterium *Coxiella burnetii*. In domestic ruminants, it is the cause of several reproductive disorders such as abortions, stillbirths, premature births, weak offspring, retained foetal membranes and infertility. In cattle, endometritis has also been reported. An inactivated vaccine, based on a phase I antigen of *C. burnetii,* is available for cattle, goats and sheep. This scientific review highlights the effects of vaccination to limit the clinical manifestations of the disease and reduce the shedding of the bacteria, thereby limiting the impact of infection for both animals and humans. The safety of the vaccine is also assessed.

**Abstract:**

Q fever is a disease caused by *Coxiella burnetii* that affects many animal species and humans. In ruminants, the disease is responsible for several reproductive disorders (such as abortions, stillbirths, premature births, weak offspring, retained foetal membranes and infertility). An inactivated vaccine based on a phase I antigen of *C. burnetii* is available for cattle, goats and sheep. This review aims to summarise the scientific literature regarding the efficacy and safety of this vaccine to control the infection in these three domestic ruminant species. Forty-five publications and one experimental veterinary thesis reporting on experimental studies, case reports, mathematical modelling and intervention studies were selected according to the PRISMA guidelines. Although some studies lack control groups or statistical analyses, for all three species, published data show that vaccination often results in a reduction in abortions and an improvement in reproductive performance in comparison with absence of vaccination. There is also evidence, including in infected herds and animals, that vaccination is associated with a reduction in bacterial shedding, both in intensity and duration in comparison with absence of vaccination. For these reasons, in case of human outbreaks, vaccination is one of the pillars of control measures. Vaccination is generally well tolerated, despite the rare occurrence of mild, transient side-effects, such as hyperthermia and reduction in milk yield.

## 1. Introduction

Coxiellosis or Q fever is an infectious disease caused by a small, Gram-negative, intracellular bacterium, *Coxiella burnetii* [1,2]. The infection affects many wild and domestic animal species, including ruminants (cattle, sheep, goats, deer), carnivores, rodents, reptiles, birds and arthropods [1,2,3,4,5,6,7]. It is also a zoonosis that can sometimes cause serious clinical signs in humans such as the outbreak in the Netherlands in 2007–2010. During this outbreak, 4026 human cases were notified. Of these, 749 required hospitalisation and 9 people died [8]. Among domestic species, it is generally accepted that domestic ruminants, either small ruminants or cattle, constitute the primary reservoir for the disease and can therefore be the source of Q fever outbreaks in humans [1,9]. A recent survey conducted in western France, in an area where Q fever is endemic in cows and the density of small ruminants is very low, reported 12% seropositivity in blood donors, suggesting that cattle can also play a role [10]. Ruminants shed the bacteria mainly in vaginal fluids, birth products (including aborted foetuses and placenta), faeces and milk. These contaminated materials can be responsible for the spread of the disease among animals and for the infection of humans. People or animals are mostly infected by the inhalation of small contaminated particles, either by direct or indirect contact with animals [1,2,11]. Ruminants can show clinical signs, the most significant being abortion, which can be epizootic in goat herds in particular [1,12]. In cattle, other clinical signs were described including endometritis and puerperal metritis [13,14], retained foetal membranes [15], pregnancy losses [16], and infertility [17]. In cattle and sheep, some studies have found an association between *C. burnetii* infection and respiratory disorders [18,19]. Additionally, an association between *C. burnetii* infection and mammary infections has been demonstrated in cattle [20,21,22] while other studies have shown an association between a qualitative or quantitative reduction in milk yield and *C. burnetii* infection in cattle and goats [23,24]. Although caused by a bacterium, antibiotic treatments have shown little effectiveness in ruminants [25,26]. Therefore, control of the disease relies on the combination of different biosecurity measures such as the systematic collection and destruction of aborted foetuses, placentas and other birth products, and vaccination [1,27].

Coxevac^®^ (Ceva Santé Animale, Libourne, France) is an inactivated, non-adjuvanted vaccine containing a purified phase I *C. burnetii* antigen of the NineMile strain. The antigen concentration is above 100 µg/mL, equivalent to 72 QF unit/mL (relative potency of phase I antigen measured by ELISA in comparison with a reference item). It was granted marketing authorization in 2010 in Europe for cattle (from 3 months of age, two subcutaneous injections of 4 mL each, 3 weeks apart, re-vaccination every 9 months) and goats (from 3 months of age, two subcutaneous injections of 2 mL each, 3 weeks apart, yearly booster). Since 2023, it is also indicated for sheep (from 4 months of age, two subcutaneous injections of 2 mL each, 3 weeks apart, new vaccination prior to each artificial insemination or mating). It is currently the only Q fever vaccine available in veterinary medicine and is commercialized in all the countries of the European Union, and in some other countries in Europe, the Middle East and North Africa. It is also available in South Africa and Canada. Interestingly, to our knowledge, this is the only vaccine currently on the market that has received a claim in cattle based on a clinical trial performed in previously infected herds [28,29,30].

The use of the vaccine in the prevention or control of Q fever has been the subject of several publications. These include articles relating to experimental studies, intervention studies, case reports and the construction of mathematical modelling tools. A review of the global control measures for Q fever in sheep, goats and cattle has recently been published [31]. However, this review focuses on the existing bibliography specifically related to the vaccine currently on the market in order to provide more comprehensive information in this area.

## 2. Materials and Methods

Bibliographic research was carried out according to PRISMA guidelines [32]. The search was restricted to articles published since 1990 to only include data relating to the vaccine currently on the market (Coxevac^®^, Ceva Santé Animale, Libourne, France). This vaccine received its first marketing authorization in 2010 but has been used in France with a temporary authorization since 2004. A 14-year safety margin was added to include data published during the vaccine’s development phase. We used the PICO (population–intervention–comparison–outcome) convention [33] with the following. Population: cattle, goats and sheep; intervention: vaccination with an inactivated phase I vaccine; comparison: none; outcomes: use of current marketed vaccine in target species. The specific question addressed by the review is as follows: “What is the effect of vaccination against Q fever with the vaccine currently on the market in cattle, goats and sheep?”

The Dialog tool (Proquest, Ann Arbor, MI, USA) was used, and five databases (BIOSIS Previews, British Library Inside Conferences, MEDLINE, CAB Abstracts and Publicly Available Content) were included. We searched for articles containing in their title or abstract all terms relating to *C. burnetii*/Q fever/coxiellosis, a term relating to vaccination and a term referring to cattle goats or sheep. Thus the query was defined as follows: ((ab,ti(q fever) OR ab,ti(coxiella) OR ab,ti(coxiellosis)) AND (ab,ti(vaccination) OR ab,ti(vaccine)) AND (ab,ti(ruminants) OR ab,ti(cattle) OR ab,ti(cow) OR ab,ti(heifer) OR ab,ti(goat) OR ab,ti(sheep) OR ab,ti(bovin*) or ab,ti(ovin*) OR ab,ti(caprin*))) AND (pd(1990–2023)). It was run in “command line” mode with the duplicate removal option enabled on the 23rd of October 2023. This bibliographical search yielded 215 results.

**Figure 1:**
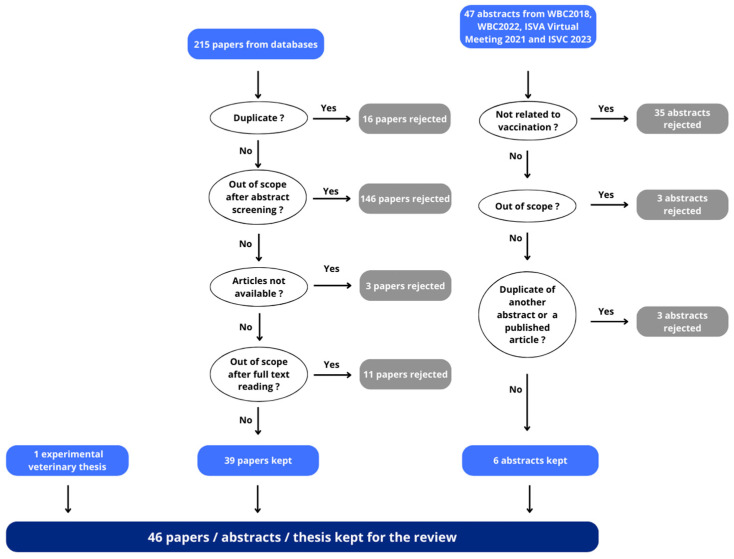
The article selection flow at each stage, showing the number of publications included and excluded at each level—from databases from conference proceedings (WBC, ISVA, ISVC).

Sixteen duplicates, in addition to those automatically removed by the Dialog tool, were manually removed. After screening, 46 articles were review articles (including 42 reviews, two theses and two scientific opinions) and therefore considered out of scope. Another 100 articles were eliminated because they were devoted to human medicine (*n* = 44), were not related to vaccination against Q fever (*n* = 35), were not related to the currently marketed vaccine (but to a withdrawn vaccine or an experimental vaccine including a Coxevac^®^ modified vaccine by addition of an adjuvant [34]; *n* = 11) or dealt with vaccination in species other than cattle, sheep or goats (*n* = 10). Four of these latter ten articles had also another reason for rejection, being not related to Q fever vaccination (*n* = 2) or because the vaccine was not licensed (*n* = 2). Finally, 53 articles were retained for full paper reading and evaluation. Unfortunately, we were unable to obtain three articles. Articles not written in English or French have been translated with the help of the https://translate.google.com, (accessed on 12 December 2023)/web application (Google Ireland Limited, Dublin, Ireland). After reading, 11 articles were eliminated because they did not deal with the effect (efficacy, immune response or safety) of the licensed vaccine (*n* = 5), were related to human medicine (*n* = 3), repeated data from a previously published study and were therefore considered duplicates (*n* = 2) or were a review (*n* = 1). We therefore retained 39 papers (Figure 1).

Abstracts from the last two international congresses dedicated to cattle and small ruminants, respectively, were also considered. Proceedings from the World Buiatrics Congress (WBC) 2018, WBC 2022, International Sheep Veterinary Association (ISVA) virtual meeting 2021 and International Sheep Veterinary Congress (ISVC) 2023 were reviewed. Note that the previous congresses were not included as we considered that older relevant data should have been published by now in a peer-reviewed journal after their presentations at the congress. Three successive screenings for each proceeding performed using the search function of Acrobat Reader (Adobe, San José, CA, USA) with the words “q fever”, “coxiella” and “coxiellosis” retrieved 47 abstracts. After reading, 35 were eliminated because they did not deal with vaccination. Of the remaining 12 abstracts, three were not related to vaccine efficacy, vaccine safety or immune response, and three were duplicates of another abstract or a published article. We therefore retained seven abstracts (Figure 1).

One student thesis [15] that did not come out of the bibliography search was included in the review because this study was carried out by the same team that conducted the study to obtain marketing authorisation for the vaccine in cattle.

## 3. Results and Discussion

Forty-six articles, conference abstracts or theses were analysed. Two articles deal with both the vaccine currently on the market and another vaccine that is not on the market because it is either experimental or no longer marketed. The distribution of articles according to source (peer-reviewed journals or congress), target species or type of study is detailed in Table 1. The studies are presented by animal species (cattle, goats and sheep), grouped within each species according to the outcome studied. Next, if available, data on immune response are presented, followed by data derived from mathematical modelling and results on safety and side effects of vaccination. To avoid repetition, the three articles concerning more than one species are discussed in the section corresponding to the species for which there was more data in the article. Therefore, one is presented in the section devoted to goats and two in that devoted to sheep.

### 3.1. Cattle

The studies related to vaccination in cattle are listed in Table 2. Interestingly, all in vivo studies in cattle, with the exception of one on vaccine safety [35], have been conducted in infected herds. Therefore, the effectiveness of the vaccine in preventing herd contamination has not been evaluated in any study.

#### 3.1.1. Reproductive Performance and Abortions

A large-scale study was carried out in 120 herds that had experienced abortions due to *C. burnetii* in the western part of France [15]. A large number of animals (*n* = 4823) were involved, and three different medical control strategies were studied (vaccination; antibiotic therapy using oxytetracycline at dry-off and/or calving; combination of vaccination and antibiotic therapy) and compared with no control measures (reference). At herd level, there was no reduction in the incidence of abortions (comparing 6 months before and 12 months after implementation of control measures) regardless of control method. At the individual level, the risk of abortion was lower, although not significantly in vaccinated animals: OR: 0.694 [0.453–1.06] (*p* = 0.09). Regarding reproductive performance, vaccinated heifers had almost half the risk of a late return to artificial insemination (AI) (from 27 to 90 days) than non-vaccinated heifers (OR = 0.538 [0.301–0.963]; *p* < 0.05). However, there was no significant difference between vaccinated or not vaccinated cows. The difference found between heifers and cows could be due to a higher ratio of infected animals before vaccination in adult than in heifers [49].

Lopez-Helguera and colleagues [17] carried out a field study in two infected farms with a total of 719 dairy cows and heifers including both seropositive (*n* = 168 cows and *n* = 11 heifers) and seronegative (*n* = 353 cows and *n* = 187 heifers) animals at the beginning of the study. The cows were randomly allocated to be vaccinated (*n* = 360) or not (*n* = 359) during the last third of gestation. The reproductive performance was assessed after calving using the conception rate at 150 days in milk. Vaccinated animals were 22% more likely to conceive than non-vaccinated animals (OR = 1.22; 1.02–1.46; CI = 95%; *p* = 0.028). If only cows that were seronegative before vaccination are considered, the results were similar (OR = 1.25; 1.02–1.54; CI = 95%; *p* = 0.030). In a clinical case report, Kneblewski and coworkers [43] also noted that the implementation of vaccination in two infected herds in Poland improved reproductive performance. In the first herd, they compared data 12 months before and 12 months after the start of vaccination in a 160-dairy cow herd. The rate of stillbirth decreased from 12.2% to 5.8%, the rate of abortion from 14.4% to 9.1%, the rate of placental retention from 26.6% to 10.3% and the rate of metritis from 18.6% to 11.0%, respectively. However, no statistical analyses were performed, and there was no control group. In the same article, the authors reported that in another herd (*n* = 250 cows), based on an interview with the farmer, the disease manifested itself in the form of reduced milk yield and respiratory disorders. After the implementation of vaccination, respiratory disorders decreased and milk production returned to normal. But no data are given by the authors for this farm. It is therefore difficult to draw a definitive conclusion.

In a control-case study [42], Garcia-Ispierto and colleagues assessed the efficacy of vaccination in the last third of gestation of cows and heifers in an infected farm. After assessment of their antibody status, heifers and cows were randomly assigned to the vaccinated group (*n* = 212; 62 seropositive and 150 seronegative) or the control group (*n* = 208; 60 seropositive and 148 seronegative). In the vaccinated group, animals received two doses of the vaccine 3 weeks apart according to the manufacturer’s instructions during late gestation (Days 171–177 and 192–198 of gestation). The same protocol was applied again the following year. After the first year of vaccination, vaccinated animals had a lower risk of early pregnancy loss during the next pregnancy than non-vaccinated animals (OR = 1.42; 1.1–2.8; CI = 95%; *p* = 0.04), regardless of their pre-vaccination serological status. After the second year of vaccination, vaccinated cows that were seronegative before vaccination were less likely (OR = 0.4; 0.2–0.8; CI = 95%; *p* = 0.02) to be sub-fertile (having had more than three artificial inseminations (AI) during the first 150 days in milk; DIM) than non-vaccinated seronegative animals. Another study conducted in Turkey in an infected herd [46] involved 575 pregnant cows (approx. 5 months of pregnancy). The animals were divided into three groups: already seropositive and not vaccinated (*n* = 174), seronegative and vaccinated (*n* = 175), and seronegative and non-vaccinated group (*n* = 226). Among pre-vaccination seronegative animals, abortion rates were 2.7% in the non-vaccinated group and 1.1% in the vaccinated group. In the seropositive group, the abortion rate was 0.6% However, these differences were not statistically significant.

#### 3.1.2. Shedding

A study [36] was carried out in France in six infected dairy herds involving 336 cows. Animals were divided randomly into three groups: vaccinated during pregnancy (between 1 and 8 months), vaccinated before pregnancy and non-vaccinated. The allocation was stratified accounting for parity and serological status before treatment (using blood ELISA) and shedding status (based on PCR on milk, vaginal mucus and faeces), and assessed twice 2 weeks apart before treatment. To be considered as non-infected, a cow had to be seronegative and a non-shedder. The authors showed that non-infected, non-pregnant animals at the time of vaccination were almost five times (OR = 0.21; 0.05–0.90; CI = 95%; *p* = 0.036) less likely to become shedders during the study period (12 months) than non-vaccinated animals. It was also noted that when the females were vaccinated during pregnancy, the risk reduction was only 10%, but the difference was not significant compared to the non-vaccinated group. In another study conducted in 77 infected dairy farms in France, Taurel and colleagues [39] evaluated the evolution of *C. burnetii* shedding in bulk tank milk (BTM). Four different strategies were randomly applied to cows within each herd: vaccination; vaccination and antibiotic treatment at drying-off and/or at calving (long-acting oxytetracycline at 10 mg/kg once or twice 2 days apart); antibiotic treatment alone; or no treatment. The outcome was considered favourable when either the prevalence of shedding animals and/or the bacterial load in BTM decreased. The probability of a favourable outcome of shedding was highest in the vaccinated groups (with or without associated antibiotic treatment) (OR = 6.59; 1.47–29.52; CI = 95%; *p* = 0.042), with the non-treated group being the reference. The authors also noted that the probability of a favourable outcome of shedding (based on negative PCR on BTM) was five times lower for herds where 20 to 80% of animals were vaccinated initially (OR = 0.20; 0.06–0.65; CI = 95%; *p* = 0.01) compared with herds where the vaccination rate was higher than 80% (i.e., vaccination of all cows and heifers). In another study [38], the same team assessed the efficacy of vaccination to prevent the shedding of vaginal mucus at calving. Calving cows (*n* = 883) in 22 herds in France were involved. In each herd, cows were randomly assigned to vaccinated groups (*n* = 348 after AI and *n* = 87 before AI) or a control group (*n* = 448). The ratios of shedder animals at calving were not significantly different between groups (21% in vaccinated group after AI, 14.9% in vaccinated group before AI and 17.0% in control group, *p* = 0.35). However, vaccinated cows were at a lower risk of being medium or high shedders (10^2^–10^4^ bacteria/mL, more than 10^4^ bacteria/mL, respectively) at calving than non-vaccinated cows (*p* = 0.03). The difference was even higher when the cows were vaccinated before AI [38]. The results are detailed in Table 3. Taken together, these studies suggest that vaccination should be given preferably before AI.

Tutusaus and colleagues [40] compared the evolution of shedding in vaginal fluids (or placenta at parturition), milk and faeces between cows vaccinated during late pregnancy (Day 171–177 with second injection 21 days later; *n* = 78) and cows not vaccinated (controls; *n* = 78). Both pre-vaccination seronegative (*n* = 42 in the vaccinated group and *n* = 48 in the control group) and seropositive (*n* = 36 in the vaccinated group and *n* = 30 in the control group) animals were included in the study. Shedding was assessed before vaccination, at parturition and then weekly for 5 weeks, as well as once at 3 months postpartum. Overall, the authors noted no difference in the proportion of animals shedding by any route between vaccinated and non-vaccinated animals. However, no information is given on the relative proportion of the different routes, the serological status of the cows before vaccination, or the evolution of the quantity of bacteria shed. Indeed, vaccination is more effective in animals that have not been previously exposed to the disease [36].

In another field study in an infected dairy farm, Piñero and colleagues [41] assessed the shedding of *C. burnetii* by cows through milk and uterine fluids, and the environmental contamination over two years. All the non-pregnant animals from 3 months of age were vaccinated according to the manufacturer’s instructions. The pregnant animals at the beginning of the study were vaccinated after calving. Therefore, no control group was included in this study. The study showed that two years after the first vaccination, none of the young animals (first and second calving) were shedders. According to the authors, vaccination and progressive culling of shedder cows due to the natural turnover of the herd effectively reduced infection burden and horizontal transmission of the infection was no longer occurring at a significant level at the end of the study. Regarding the environmental contamination, the DNA of *C. burnetii* was still present in the slurry 18 months after the implementation of vaccination, whereas dust surfaces and air became negative 6 months after the first vaccination. Finally, at the end of the study, no environmental contamination remained.

In a case report, Böttcher and colleagues [47] reported the vaccine’s efficacy in reducing the number of animals shedding in vaginal mucus and milk over 5 years. At the beginning of the study, 9 out of 59 cows shed vaginally. Four years later, no cow was shedding. For milk, the number of shedders decreased from 7 out of 191 to 3 out of 225 cows. However, no control group was included, and no statistical analysis was given. The authors also indicate that vaccination is ineffective in chronic shedders. But the vaccination protocol did not include annual booster doses after the second calving, as is recommended [30].

#### 3.1.3. Other Impacts of Vaccination

A survey [44] has been carried out in Germany among farmers whose herds had been diagnosed as infected with Q fever and who had been implementing vaccination for at least three months. Fifty farmers, with a total of 10,408 cattle, were interviewed about the benefits of vaccination. Most (84%; *n* = 42) of the farmers interviewed indicated that vaccination had improved the overall health of their herd. For 50% of farmers (*n* = 25), vaccination had a positive effect on milk production. This was confirmed by data on six farms. In these herds, average production increased significantly by 2.55 kg/cow/day when comparing production before and after vaccination (*p* = 0.001). Conversely, on three farms, no effect on production could be demonstrated during the first year of vaccination, but this effect was visible after the first booster the following year. Fifteen farmers thought that the major problems on their farms were improved as soon as the primary vaccination was carried out. This included reduced abortions, mastitis and retained placenta. Several farmers also mentioned an overall improvement in fertility. However, five farmers thought that vaccination did not bring any improvement. Overall, 68% of farmers planned to continue vaccination, and 16% would do so on the advice of their vet. Interestingly, several farmers stated that they would continue to vaccinate because of the risk to themselves (i.e., the zoonotic risk). In a survey, the information given by farmers is obviously subjective. In addition, no farmer with an infected but non-vaccinated herd was included. Therefore, drawing any scientific conclusions from this survey is difficult. Nevertheless, this study seems to confirm the clinical efficacy of cattle vaccination against Q fever at the level of the end user.

#### 3.1.4. Mathematic Modelling

In 2011, Courcoul and colleagues [37] proposed a stochastic epidemic model based on individuals, coupled with herd demographic dynamics. This model was used to evaluate over time the prevalence of shedders, environmental contamination with *C. burnetii* and the number of abortions based on three vaccination strategies (annual vaccination of cows and heifers for 10 years; annual vaccination of cows and heifers for three years; and annual vaccination of heifers only for 10 years). The three strategies were compared with no intervention (no vaccination). All three vaccination strategies resulted in a significant reduction in abortion rates, environmental contamination and the number of shedders. However, in the case of vaccination of heifers only, this reduction was delayed from 9.2 to 9.9 months, depending on the parameter assessed, compared with vaccination of the whole herd. If vaccination is stopped after 3 years, the model shows that all three parameters increase again and that the benefit of vaccination obtained in the first three years is compromised.

Based on this model, Asamoah and colleagues [45] proposed another model to assess the impact of vaccination, hygiene (biosecurity measures) and culling or isolation of infected animals in the case of a Q fever outbreak in dairy cattle. Not surprisingly, the combination of the three measures showed the best efficacy. However, culling is not an acceptable measure in most cases. The model shows that in the absence of culling/isolation measures, the implementation of biosecurity measures alone does not reduce the impact of the disease, either on the number of asymptomatic infected cattle or on environmental contamination. The authors point out that the total absence of control measures or the implementation of biosecurity measures alone gives similar results. The model also shows that effective control of a Q fever episode requires vaccination of at least 80% of animals as soon as possible after the onset of the infection (during the first 20 to 80 days). The authors theoretically evaluated the costs of different control measures and compared them with the cost of the disease when no control measures are implemented. Their analysis concluded that implementing all three measures (vaccination, biosecurity and culling/isolation) reduces the economic impact of the disease by 97.8%, by 95.9% with vaccination and biosecurity, and by 63.0% if only biosecurity and culling/isolation are implemented. This confirms the essential role of vaccination in managing Q fever. In conclusion, the authors state that their model shows that eradication of the disease is not fully possible. However, the combination of placenta management (biosecurity) and vaccination can significantly limit the spread of the disease in the animal population.

Raboisson and colleagues [48] developed a bioeconomic model to reproduce the effect of vaccination on an infected herd over 3 years. The economic benefit of vaccination was estimated: for a herd of 100 cows with a low prevalence of the disease (20%), the benefit over three years is estimated at EUR 3169. If the prevalence is higher (40%), this benefit rises to EUR 11,937.

#### 3.1.5. Safety and Side Effects

In the survey conducted by Lehner and colleagues [44] across 50 cattle farms, half of the farmers did not notice any side effects of vaccination, while 18% of them reported classical signs (i.e., slight weakness lasting less than 2 days, slight elevation of rectal temperature) which usually occur after vaccination regardless of the type of vaccine. About 20% of the farmers noticed a slight and transient drop in milk yield. Most of the time, the side effects disappeared within 2 weeks. Occasionally, some farmers noticed a local reaction (*n* = 2) or an increase in the milk Somatic Cell Count (*n* = 1). These findings are in accordance with the number of adverse events declared to the authorities by users. For example, during the temporary authorisation period of the vaccine in France in 2005–2006, 158 adverse events were reported for 115,562 doses injected (i.e., 0.137%).

An experimental study [35] has also demonstrated a slight, transient increase in rectal temperature, as well as an effect on milk yield. Vaccinated cows (*n* = 246) produced less milk during the first 7 days following the first vaccination injection than non-vaccinated cows (*n* = 252): vaccinated, 26.8 ± 0.39 L per day; not vaccinated, 28.2 ± 0.44 L per day; *p* = 0.033). However, it was noted that this impact was more marked for high-producing cows not previously vaccinated against *C. burnetii*. The authors conclude that vaccinating animals first while they are heifers should protect them from most side effects when they later receive a booster vaccination during lactation.

Others found that vaccination during the last third of gestation had no side effects [17,40]. In a study involving both vaccinated pregnant and non-pregnant animals, Guatteo and coworkers [36] observed neither local reactions nor general side effects including in cows vaccinated in the last month of pregnancy.

All these data are in accordance with the low number of adverse effects of Coxevac^®^ in cattle reported in the European Database of suspected adverse drug reaction report (108 cases since 2008). In some cases, several disorders have been declared, the most common being systemic disorders (*n* = 56), followed by disorders related to the reproductive system (*n* = 37), the mammary gland (*n* = 32) or the application site (*n* = 30) [50].

### 3.2. Goats

The studies related to vaccination in goats are listed in Table 4. Only one in vivo study [51] assessed the efficacy of the vaccine to prevent contamination of goats in a non-infected environment prior to vaccination. However, another one [52] aimed to assess the efficacy of control measures at a country level. It therefore included data on non-infected herds.

#### 3.2.1. Abortions and Shedding

In an experimental study [51], the efficacy of vaccination with Coxevac^®^ (*n* = 16) or a bivalent inactivated vaccine (*Chlamydophila abortus* and *C. burnetii* phase II; *n* = 15) were evaluated in naïve goats. After vaccination, goats were synchronized, inseminated and challenged with a subcutaneous injection of 10^4^ *C. burnetii* (strain CbC1 bacteria) at 84 days of gestation. A control group was not vaccinated but challenged (*n* = 12), and another control group was neither vaccinated nor challenged (*n* = 27). The abortion rate was 6% in the vaccinated group with Coxevac^®^, 87% in the vaccinated group with the bivalent phase II vaccine, 75% in the non-vaccinated, challenged group, and 15% in the non-vaccinated, non-challenged group. The abortion rates were not significantly different between the Coxevac^®^ and the non-vaccinated/non-challenged group. In opposition, the difference was significant between the Coxevac^®^ group and the two other challenged groups (Kruskal-Wallis test; *p* < 0.01). This highlights the efficacy of vaccination in preventing abortions when implemented in naïve animals. *C. burnetii* was detected in the placentas of all non-vaccinated, challenged goats, in 14 of 15 (93.3%) of bivalent phase II vaccinated goats and in 6 of 16 (37.5%) Coxevac^®^ vaccinated goats. The bacterial load per placenta was reduced by 5 to 7 log_10_ in the Coxevac^®^ vaccinated group (0.89 +/− 1.05 log_10_) compared with the non-vaccinated group (7.25 +/− 1.14 log_10_) and the bivalent phase II vaccinated group (6.27 +/− 1.28 log_10_). Shedding of *C. burnetii* was also assessed in milk, vaginal mucus and faeces. In vaginal mucus, 100% of challenged, non-vaccinated goats, 93.3% of bivalent phase II vaccinated goats and 37.5% of Coxevac^®^ vaccinated goats shed the bacteria. Finally, no Coxevac^®^ vaccinated goats shed *C. burnetii* in milk, whereas all challenged, non-vaccinated and 93.3% of bivalent phase II vaccinated goats shed *C. burnetii* in milk. The duration of shedding is reported in Table 5.

A clinical case was reported by Hurtado-Preciado and coworkers in Spain [60] where *C. burnetii* DNA was found by PCR in a foetus after an abortion outbreak in a herd with 40 lactating goats. All the goats older than 3 months of age were vaccinated (two 2 mL subcutaneous injections 3 weeks apart). According to the authors, abortions ceased as early as one week after the first injection. However, this conclusion should be considered with caution, as vaccination is not a therapeutic solution and no control group was included in this case report.

Sting and coworkers [57] described an acute episode of Q fever on a goat farm (63 dairy goats at the beginning of the study) in Germany. The event manifested itself as abortions at the end of the kidding campaign and clinical signs in farm workers. About 6 months after this episode, vaccination was administered to 120 goats 4 weeks before breeding, and shedding was monitored by analysis of vaginal swabs within 2 weeks after parturition. No control group was included in this study. Six months after vaccination, out of 48 goats sampled, 2 had a bacterial load per swab greater than 10^4^, 21 lower than 10^4^, and 25 were negative. One year later (i.e., 6 months after the booster injection), out of 87 goats sampled, 79 were negative, while 8 were positive but with bacterial loads of less than or equal to 50 bacteria per swab. Another study [56] was carried out in three goat herds that had experienced waves of Q fever-related abortions. Vaginal shedding (swabs) and clinical signs were monitored. One of the interesting aspects of this study was that the animals were separated into eight groups for statistical analyses. Susceptible animals (i.e., seronegative and non-shedding) were considered along with animals already infected. For each group, primiparous and multiparous animals were evaluated independently. Finally, for each of the four groups defined above, half the goats were vaccinated before pregnancy and the other half formed the non-vaccinated control group. The distribution of animals is shown in Table 6.

The authors reported an overall effect of vaccination on the reduction in abortions (16/427 in vaccinated groups and 22/449 in the control groups) and stillbirths (4/427 in the vaccinated groups and 6/449 in the control groups). However, due to the small number of abortions and stillbirth, the differences were not statistically significant. The results also showed that in highly infected environments, while vaccination did not modify the risk of being shedder, it was associated with a significant reduction in the quantity of bacteria shed in vaginal mucus (−0.89 log_10_ on average, *p* < 0.05). However, the results must be interpreted with caution, as only 50% of the animals in each herd were vaccinated, and they remained in contact with non-vaccinated animals. Therefore, a probable underestimation of vaccination efficacy is possible. Indeed, it has been shown in dairy cattle that a minimum of 80% of the animals in the herd must be vaccinated to reduce the circulation of *C. burnetii* in the herd [39]. An earlier study [53] in a goat herd with storm abortions (72% of abortions in four months) also showed that vaccination of already-infected animals did not eliminate vaginal shedding but did significantly reduce it. The proportion of highly shedding animals (>10^6^ per vaginal swab) was 13% in non-vaccinated goats and 4% in vaccinated goats. Conversely, the proportion of low-level shedders (<200 bacteria per vaginal swab) was 4% and 24% in non-vaccinated and vaccinated goats, respectively (*p* = 0.02). These results are consistent with what was found in an experimental study [64]. Vaccinated (*n* = 14) and non-vaccinated goats (*n* = 7) were challenged subcutaneously with a heterologous field strain of *C. burnetii* (CbC1) one year after the second injection of primary vaccination. Shedding was monitored in faeces and vaginal mucus from 14 days after challenge until 35 days after parturition or abortion. Shedding in milk was assessed from the day of parturition/abortion for 35 days. The proportion of shedders (in faeces) was 100% and 28.6% (*p* < 0.003) in the control and vaccinated groups, respectively. For vaginal mucus, the proportion of shedders was 100% and 25% in the control and vaccinated groups, respectively (*p* < 0.002). For shedding in milk, the values were 100% and 16.7% in the control and vaccinated groups, respectively (*p* < 0.0002). The mean level of shedding per mL in vaginal mucus was 5 × log_10_ lower in the vaccinated group (<2 log_10_/mL) than in the non-vaccinated group (between 7.3 log_10_ and 8.7 log_10_/mL, depending on the sampling day). This reduction was 4 × log_10_ per gram in faeces (<2.6 log_10_/mL in the vaccinated group and 6.5–6.6 log_10_/mL in the control group). The same decrease was found in shedding in milk (<2 log_10_/mL in the vaccinated group and 5.5 log_10_/mL in the control group). Regarding abortion rate after the challenge, five out of seven goats in the non-vaccinated group aborted (71.4%) compared to only three out of fourteen in the vaccinated group (21.4%; *p* = 0.04).

In another study, Toledo and colleagues [67] showed that vaccination reduced the proportion of goats shedding *C. burnetii*. Six animals were included. Prior to vaccination, the DNA of the bacteria was found in the faeces and nasal cavities of all goats. Four goats were also positive for vaginal mucus and one was positive for milk. Two months after vaccination, vaginal swabs, faeces and milk were negative in all animals. As for nasal cavities, only two goats were positive. However, this study, involving only six infected animals without a control group, does not allow any definitive conclusions to be drawn. Moreover, the detection of *C. burnetii* DNA in nasal cavities should be interpreted with caution as it may result from either individual respiratory shedding or environmental contamination.

Bauer and coworkers [65] studied the dynamics of *C. burnetii* on three goat farms in Germany following vaccination. In two farms, all the animals were vaccinated every year, while in the third herd, only young offsprings were vaccinated yearly. There was no control group. All three farms had experienced clinical signs of Q fever (abortions, weak kids, stillbirths). The study focused on vaginal shedding, milk shedding (monthly BTM analyses) and environmental contamination. In all three herds, one year after vaccination, there was a reduction in the number of goats shedding the bacteria, and the bacterial load of positive vaginal swabs was lower. Regarding shedding in milk, two herds were intermittently positive for BTM, but, when positive, bacterial loads were low. Environmental contamination was progressively reduced, but after three years, around half the dust samples were positive. This proportion was higher in the herd that did not continue vaccination on adult goats. But data from dust samples should be interpreted with caution since *C. burnetii* DNA can be detected for an extended period and the viability of the bacteria has not been assessed in this study. It is therefore possible that the DNA detected, or at least part of it, is old (from the original outbreak) and not from bacteria recently shed by the animals. However, all these data are in agreement with a previously published mathematical model [63] highlighting the importance of continuing vaccination for a minimum of 6 years (see Section 3.2.3).

Van den Brom and colleagues [62] summarized the effectiveness of the measures put in place to tackle the major outbreak of Q fever that occurred in the Netherlands from 2007 onwards. Their study was based on the presence of *C. burnetii* in BTM. The authors showed that compulsory vaccination of farms in addition to other measures (like culling of pregnant animals) reduced the risk of shedding of the bacteria in BTM by a factor of three after one year. The risk was then reduced by a factor of 10 after 2 years of vaccination (OR = 0.1; 0.1–0.2; *p* < 0.001), 30 after 3 years of vaccination (OR = 0.03; 0.02–0.05; *p* < 0.001) and 100 (OR = 0.01; <0.01–0.02; *p* < 0.001) in years 4 and 5 after vaccination. The authors also indicate that no abortion related to *C. burnetii* occurred in small ruminants after the implementation of compulsory vaccination. Similar results were found in Belgium [59]. The introduction of compulsory vaccination on 14 farms with infected goats led to a reduction in the shedding of the bacteria in BTM based on the PCR Ct value. Although no statistical analysis was given, the authors noticed that this reduction was continuous for around 15 months, after which shedding increased again slightly. However, as the Coxevac^®^ duration of immunity is 12 months in goats [64] and no booster injections were given, it is likely that residual immunity after 15 months was not sufficient to prevent shedding by animals.

On another farm in the Netherlands, van den Brom and colleagues [58] found that 5 goats out of a total of 350 resumed shedding *C. burnetii* in their milk more than two years after vaccination. Although pre-vaccination serology results were only available for one animal, these five goats were likely infected before vaccination and that, consequently, shedding may have continued since then. Interestingly, after the necropsy, PCR analyses were performed on several tissues of these five animals: circulatory and hematopoietic system, reproductive system (including mammary gland), urinary tract, digestive tract and respiratory tract, as well as vaginal swabs, uterine content, urine, blood and milk. Only milk and mammary gland tissue were positive. However, immunohistochemical analysis on mammary gland tissue failed to identify *C. burnetii* and histology showed no inflammatory process. This raises the question of whether live *C. burnetii* were still being shed by animals or whether only residual genetic material existed from an earlier infection. However, Bauer [66] reports the case of a chronically infected goat in which *C. burnetii* was detected in mammary tissue by immunohistochemistry and fluorescence in situ hybridization.

Hogerwerf and colleagues [55] carried out a study on 13 infected dairy farms (12 goat farms and one sheep farm) in the Netherlands. Vaccination was carried out on seven goat farms. The study took place in early 2010 during the period when pregnant goats and ewes on infected farms were being systematically culled as part of the Q fever eradication program. This made it possible to collect the uterine contents of 957 pregnant animals at necropsy (vaccinated *n* = 470; non-vaccinated *n* = 487). Vaginal mucus (vaccinated *n* = 404; non-vaccinated *n* = 420) and milk (vaccinated *n* = 248; non-vaccinated *n* = 242) were also collected. Results showed that for vaccinated females, 0.43% of uterine samples, 30% of vaginal swabs and 4% of milk samples were positive for *C. burnetii*. For non-vaccinated animals, the positivity accounted for 26%, 76% and 33% for uterine samples, vaginal swabs and milk samples, respectively. The odds ratio of having DNA of *C. burnetii* in uterine fluid or in milk was two times lower in vaccinated animals than in non-vaccinated animals (OR = 0.49; 95% CI 0.34–0.70; *p* < 0.05 and OR = 0.54; 95% CI 0.39–0.75; *p* < 0.05 respectively). Regarding vaginal mucus, the risk of shedding was three times lower in vaccinated animals than in non-vaccinated ones (OR = 0.34; 95% CI 0.28–0.42; *p* > 0.05). These data also take into account results from the non-vaccinated ovine flock. Since sheep shed less bacteria than goats [68,69,70], it is likely that the proportion of positive samples, whatever the route of shedding, would have been even greater if the authors had only considered goat flocks.

On a population scale, a study of the mandatory vaccination protocols implemented after the diagnosis of Q fever on sheep and goat farms in Belgium showed that rigorous application of the vaccination protocol led to a significantly greater and faster reduction in shedding (in BTM) than when the vaccination protocol was not compliant (intermittent, non-synchronized vaccination). The same study showed that vaccinated naive herds (defined as being both seronegative prior to vaccination and PCR negative for BTM) remained free of the disease, which was confirmed by the fact that the BTM remained negative [52].

#### 3.2.2. Other Benefits of Vaccination

In the case report detailed above [60], the authors also noted that milk production in the vaccinated goats (*n* = 40) doubled after the first vaccination (from 20 L to 40 L per day). Milk production then decreased during the week following the second injection (to 33 L per day), before increasing again to 40 L per day one week later. However, no hypothesis was put forward to explain this effect.

#### 3.2.3. Mathematic Modelling

Based on data from the major Q fever outbreak that occurred in the Netherlands between 2007 and 2010, a mathematical model was built [63] to assess the effectiveness of different disease control methods on goat farms. Not surprisingly, preventive vaccination prevents the onset of the disease on farms. When farms are already contaminated, only the implementation of vaccination, either following an abortion storm or the discovery of positive BTM, leads to the absence of abortions and infected animals, as well as the disappearance of environmental contamination. The other measures evaluated, i.e., the elimination of pregnant females following an abortion storm, the test and cull strategy (culling of positive animals) or a breeding ban, do not lead to the disappearance of the disease from the farm, which is also not achieved in the absence of measures. The model also shows that vaccination must be extended for at least 5 to 8 years, depending on the type of breeding, to eradicate the disease.

The same model was used to assess the cost of the different strategies. Taking no action at all was the least costly solution per year (on average, between EUR 2778 and 3330 for a 600-goat farm, depending on the breeding management style) but the model is based on the assumption of a single introduction of an infected pregnant animal in the herd. The authors also highlight that the disease cannot be eradicated by this measure. Preventive vaccination is the second, less expensive measure (between EUR 4012 and 4048). This allows the disease to be eradicated within an average of 2 years. Vaccination in an infected environment (after an abortion storm or a positive result in BTM) comes next (between EUR 4332 and 4883). Finally, all other measures are more expensive (between EUR 8397 and 59,189). Notably, culling pregnant females after an abortion storm costs 10 times more than preventive vaccination. Therefore, preventive vaccination appears to be the best strategy on the basis of the cost and the time required to eradicate the infection in a herd [61].

#### 3.2.4. Safety and Side Effects

In Arricau-Bouvery and colleagues’ experimental study [51], no local or systemic reactions were observed within 8 days of vaccination. In a field case [57], 120 goats were vaccinated twice 3–4 weeks apart followed by a booster at 12 months. A few local reactions and a slight rise in rectal temperature in one animal were noted. The general health of the animals was not affected. This is consistent with the observations of Hurtado-Preciado and colleagues [60]. In their case report, they mention a few cases of local reactions that disappeared spontaneously after 6–7 days, and that no animal showed adverse systemic signs. However, based on the European Database of suspected adverse drug reaction report, the incidence of adverse effects of Coxevac^®^ is very low: 218 cases have been notified in goats in Europe since 2008. In some cases, several disorders can have been declared, the most common being systemic (*n* = 181), followed by disorders related to the mammary gland (*n* = 110), the reproductive system (*n* = 28) or the application site (*n* = 22) [50].

It has also been shown [54] that DNA from the vaccine strain of *C. burnetii* can be found in milk up to 9 days after injection. Therefore, a false-positive PCR result in milk samples is possible in the days following vaccination.

### 3.3. Sheep

The studies related to vaccination in sheep are listed in Table 7. Like for goats, only one experimental study [71] assessed the efficacy of the vaccine to prevent contamination of ewes in a non-infected environment prior to vaccination and experimental challenge. It is important to note that the majority of studies were carried out before marketing authorisation was granted for sheep. As a result, in many studies, the protocol recommended by the manufacturer since 2023 has not been followed, particularly with regard to the vaccine dose.

#### 3.3.1. Abortions

The clinical efficacy of Coxevac^®^ was demonstrated by an experimental challenge in pregnant ewes [71]. Animals were infected by a subcutaneous injection of 10^6^ infective mouse doses (IMD) of *C. burnetii* 102 days after mating, corresponding to 130 days after the end of the vaccination protocol (two injections 21 days apart; *n* = 6). Non-vaccinated ewes (*n* = 6) and ewes vaccinated with an experimental phase II vaccine (*n* = 6) were also challenged in the same way. No abortions attributed to *C. burnetii* challenge were observed, and lamb survival was non-significantly different between the vaccinated and unvaccinated groups. The number of normal pregnancies, defined as all lambs from the same ewe born healthy and still alive at the end of the study, was significantly higher in the vaccinated groups than in the unvaccinated group despite the limited number of animals per group (*p* = 0.008).

#### 3.3.2. Shedding

In the study described above [71], *C. burnetti* shedding in faeces, vaginal fluids and milk was monitored from lambing or abortion until post-mortem examination, which took place around 4 weeks after abortion or lambing. With the exception of two ewes that temporarily shed the bacteria only in vaginal fluids (one and three days after lambing), none of the vaccinated ewes were shedders. In contrast, in the non-vaccinated group, at each sampling date (day of lambing—Day 0, Day 1, Day 2, Day 3 and Week 1, Week 2 and Week 3), all ewes (*n* = 6) shed the bacteria by at least one route. Interestingly, at Week 3, all the ewes were shedders in milk, 5 were shedders in vaginal mucus, and 3 were shedders in faeces. Therefore, three ewes shed the bacteria by all three routes.

Another team [72] studied two highly infected flocks with 266 and 269 ewes, respectively. The abortion rate was 6.3% and 5.2% for each flock, respectively, and the seroprevalence was 35.7% and 43.8%, respectively. Three-quarters of the ewes and half of the yearlings were vaccinated, keeping some non-vaccinated animals to form a control group in each flock. Abortion rates in the next lambing season were 1.9% and 1.8%, respectively, for each flock, and the bacterial load in vaginal swabs fell between the two lambing seasons (6.5 × log_10_ vs. 3.4 × log_10_ for flock 1, and 7.3 × log_10_ vs. 2.9 × log_10_ for flock 2). However, the proportion of ewes shedding vaginally and the bacterial burden shed were not significantly different between the vaccinated and the non-vaccinated groups. It is possible to assume that the bacterial contamination of the environment decreased significantly as most of the animals were vaccinated. Therefore, the risk of infection for non-vaccinated animals also decreased. Another possible explanation is a natural reduction in the number of shedding ewes, as demonstrated by Álvarez-Alonso and colleagues [82]. The authors concluded that optimal vaccination results in heavily infected flocks may not be obtained in the short term and require earlier vaccination of replacement lambs.

A 4-year vaccination field study [73] in an infected flock showed a progressive reduction in the number of shedding animals and the bacterial load in the vaginal mucus, milk and faeces of shedders. Some animals were kept unvaccinated and served as a control group. By the third year, no ewes were shedding *C. burnetii* in vaginal mucus or milk. Faeces were only tested in the first 2 years, but in Year 2, 4.0% of ewes and yearlings were shedders, compared with 72.7% in Year 1. Regarding environmental contamination, all aerosol samples were negative in Year 4, but dust samples (3/18) taken from surfaces in the animal premises tested PCR-positive for *C. burnetii*. The study did not reveal any effect on the abortion rate, but it was found that toxoplasmosis was also present in the flock.

Similar results [75] were found in another heavily infected flock monitored for 4 years. In the first year, the authors preferred to treat with antibiotics rather than vaccinate because most of the ewes were in late pregnancy. The antibiotic treatment showed no effect in reducing the shedding of *C. burnetii*. Vaccination was introduced from Year 2 onwards. Then, 75% of the animals were vaccinated and the remaining 25% were kept as unvaccinated control group. The global proportion of shedding animals fell from 97.5% to 28.1% (*p* = 0.0001) in the first year after vaccination. In Years 3 and 4, the proportion of shedders was 2.2%. There was no significant difference between groups in the percentage of shedders. Regarding the bacterial load excreted, the untreated animals tended to shed slightly more bacteria than vaccinated animals (*p* = 0.058). No conclusion could be drawn on the reduction in the number of abortions, as the herd was also infected with Border disease virus. However, once vaccination had been implemented, analyses carried out on ewes that had aborted (foetal membranes, vaginal swabs) or their foetuses did not show the presence of *C. burnetii* DNA.

These results are consistent with another field study without a control group carried out in three infected flocks [74]. In the first year, 75% of adult ewes and 50% of the replacement ewes were vaccinated. In Years 2 and 3, all the ewes and the replacement ewes were vaccinated. One year after vaccination began, the abortion rate decreased in two of the flocks (4.5%, 1.9% and 1.8% in Year 1 vs. 3.0%, 6.3% and 5.2% in Year 0 for flocks 1, 2 and 3, respectively). In Year 2, the abortion rate was 1.9%, 1.5% and 2.7% for the three flocks, respectively. After the second year post-vaccination, the percentage of shedders after parturition decreased considerably, with shedding almost disappearing during the third year after vaccination. Indeed, only 2% of the animals were still shedding *C. burnetii* in vaginal fluids in the three flocks, while 27%, 49% and 12% of the animals in flocks 1, 2 and 3, respectively, were shedders before the implementation of vaccination. However, the bacteria were still present in the environment. Despite the absence of a control group in this study, the results of this study suggest that vaccination must be continued for more than 3 years to move towards disease eradication.

In a mixed sheep–goat flock/herd that had experienced a major abortion episode linked to *C. burnetii* in both species, antibiotic treatment (oxytetracycline) and vaccination were administered to pregnant animals (*n* = 243) [76]. Bacteraemia and shedding in faeces and vaginal mucus were assessed by PCR. Before the implementation of vaccination, bacteraemia was detected in 6% of the ewes, in 10% before the second vaccine injection, in only 2% 4 months later, and in none at 8 months post-vaccination and until the end of the study (14 months). The statistical analysis confirmed a significant decrease in bacteriemia after vaccination (*p* = 0.001). Regarding faecal shedding, 23 ewes (9.5%) were positive at the time of vaccination, a single ewe 8 months later and none at 12 months. Two months later, three animals again shed in faeces. Overall, the data related to faecal shedding were not statistically different (*p* = 0.34). For vaginal shedding, 21 animals (9%) were positive at the time of vaccination, and then 11 (5%) and 1 (0.4%) at 1- and 4-months post-vaccination, respectively. Samples taken from 8 months up to the end of the study were all negative. Although there was no control group, antibiotic treatment was given in addition to vaccination, and animals were not vaccinated in accordance with the manufacturer’s instructions, the authors pointed out that vaccination was effective in reducing shedding during the outbreak of Q fever [76].

Also in an infected environment, another study [79] aimed to assess the vaccine’s efficacy on vaginal shedding and abortion rates by comparing two flocks, one with vaccination and one without, over 2 years. Although the initial seroprevalence was different between the two flocks, and *Chlamydia* infections were also present, this study did provide some interesting information on vaginal shedding. Ninety-seven ewes were tested in each flock. In the non-vaccinated flock, more ewes shed the bacteria after lambing or abortion (12.4% vs. 2.1% in the vaccinated flock; *p* < 0.02). At the herd level, no significant differences were found in abortion and stillbirth rates, but these were low on both farms. In a previous study [78], the same authors evaluated the reduction in shedding and the number of abortions in three flocks where Q fever was diagnosed by PCR on the placenta or on a pool of foetal organs following abortions. Vaccination was implemented rapidly, even though most ewes were still pregnant. The following year, no abortions were reported in any of the three flocks. In one flock, a reduction in *C. burnetii* shedding in vaginal mucus was observed as early as 3 weeks after the second injection of the primary vaccination, whereas it was more gradual (1 year) in the other two flocks. The same team [69] carried out a non-controlled study on a farm contaminated with Q fever. In this farm with sheep, goats and cattle, vaccination was implemented on all animals and shedding by sheep and goats was monitored using vaginal, rectal and nasal swabs, as well as milk samples. Environmental contamination was assessed on windowsill dust and in bedding straw. For vaginal swabs, 1 year after vaccination, the proportion of positive samples fell from 97.2% (*n* = 35/36) to 2.9% (*n* = 1/35) for sheep, (*p* < 0.05), and from 100% (*n* = 22/22) to 42.9% (*n* = 3/7) for goats (*p* < 0.05). Results were similar for rectal swabs: from 97.3% (*n* = 36/37) to 5.7% (*n* = 2/35) for sheep (*p* < 0.05) and from 97.3% (*n* = 36/37) to 57.1% (*n* = 4/7) for goats (*p* < 0.05). In milk, at the start of the study, all goats (*n* = 12/12) and 80% (*n* = 24/30) of ewes shed *C. burnetii*, whereas 1 year after vaccination, no animal excreted *C. burnetii* (*p* < 0.05). Finally, all nasal swabs of both sheep and goats were positive at the start of the study. One year later, 20% of sheep (*n* = 7/35) and 57.1% of goats (*n* = 4/7) were still positive. The authors also graphically presented the results of the bacterial load in vaginal, rectal and nasal swabs as well as in milk. It appears that positive samples one year after vaccination had a lower average bacterial load than positive samples at the start of the study, but no precise values were given. Regarding environmental contamination, there was a marked decrease over time. In particular, bedding straw samples from the cattle herd were negative from 13 months after the start of the study, i.e., 12 months after the second primary vaccination injection. For samples from sheep and goat farms, the decrease in the bacterial load of bedding was around 3 to 4 log_10_ over the same period.

Böttcher and coworkers [80] followed a 650-sheep flock infected by *C. burnetii* for 10 years, where vaccination was implemented after the diagnosis of Q fever contributing to abortions. Each year, most gimmers (around 120 animals) received a primary vaccination; 20 gimmers were not vaccinated and were used as controls. Adult ewes were not vaccinated. Monitoring was carried out using vaginal swabs (taken at lambing), nasal swabs and serological monitoring of non-vaccinated control animals to detect possible seroconversion linked to contamination. During the first year, the number of shedders (vaginal and nasal) did not decrease: around 20% of the animals were positive by qPCR in vaginal swabs and/or nasal swabs. Similarly, the bacterial load on swabs remained virtually unchanged. But from 18 months onwards, all swabs were negative. Regarding serological analyses, the number of control animals seroconverting fell progressively, reaching 1 animal in 20 analysed in years 3 and 4, and then no animals from year 5. Eight years after the start of vaccination, 1 vaginal swab out of 100 taken was positive, as well as 1 nasal swab out of 30 taken. Environmental testing revealed contamination of the lambing area. Although the results suggest that the disease burden was reduced, we can assume that the elimination of the bacteria on the farm was not complete or that the farm was contaminated again. This could be explained by the vaccination schedule chosen by the authors of this study: contrary to the protocol recommended by the vaccine manufacturer, only hoggets were vaccinated. This assumption is in accordance with other studies [52]. As some animals had not been vaccinated for several years, they were susceptible to new infections. In addition, although the shedding dynamics are different between cattle and sheep, a mathematical model provides relevant information [37]. This model showed that around two additional years are needed to achieve the same vaccine efficacy for clinical expression and shedding of *C. burnetii* when only heifers are vaccinated.

A study was carried out in sheep in Tunisia aimed at determining the risk factors for *C. burnetii* infection [77]. The study involved 164 animals from 110 different flocks located in the central-eastern part of the country. Based on PCR performed on vaginal swabs, milk and blood, it was shown that non-vaccinated animals were 8.8 times more likely (*p* = 0.045) to be infected with *C. burnetii* than vaccinated animals.

#### 3.3.3. Immune Response

Joulié and coworkers [70] monitored the serological response of three categories of ewes (aborted dairy females, normally lambed dairy females and suckler females) before and after vaccination. In the group of aborted ewes, most animals were highly seropositive before vaccination (8 out of 11) and remained so afterwards. However, two animals did not seroconvert after vaccination, but the authors do not explain this finding. In the group of ewes with normal lambing (*n* = 26), the immune response to vaccination was obvious, including females already seropositive before vaccination. However, the authors noted that three out of nine ewes that were seronegative before vaccination remained seronegative. No hypothesis was given to explain this observation. Finally, in suckler ewes (*n* = 20), all ewes except one seroconverted or remained seropositive. For all three categories of ewes, antibody levels remained stable until the end of the study, more than 7 months after the second injection of primary vaccination. All young lambs showed an increase in antibody levels, although this varied from one individual to another. The increase was more moderate in lambs from seropositive dams. However, since the lambs received their first vaccine injection at around 3 months of age (manufacturer’s recommendation is 4 months in sheep, while it is 3 months in goats and cattle), an interaction with antibodies in colostrum cannot be ruled out.

In an experimental study already cited above [71], antibody (IgG) levels after two injections of Coxevac^®^ or the experimental phase II vaccine increased sharply above the ELISA kit’s positivity threshold (S/P = 40%) but fell below this value 49 or 63 days after the second injection of Coxevac^®^ or the experimental vaccine, respectively. However, the S/P value remained at around 20% in vaccinated animals, which is higher than the value for non-vaccinated animals (0%). Interestingly, in the same study, non-vaccinated ewes experimentally challenged with an injection of 10^6^ IMD of *C. burnetii* did not show an immune response above the kit threshold. However, the *C. burnetii*-specific antibody response in the unvaccinated group was significantly lower across the whole study period (224 days) compared to the vaccinated groups (*p* < 0.001). Therefore, it appears that the qualitative interpretation of the results of the ELISA kit used does not allow an estimation of the immune response. This is in line with a study that showed a different sensitivity of ELISA kits for the diagnosis of Q fever [83]. In another study, Bauer and colleagues [81] investigated the kinetics of the immune response induced by vaccination with Coxevac^®^ in young sheep from a flock not infected with Q fever. The study only involved a small number of animals (*n* = 18). Of these, 12 were vaccinated, either with 1 mL (*n* = 6) or 2 mL (*n* = 6), and 6 received saline as a control. The vaccination protocol consisted of two injections 21 days apart. As early as 21 days after the first vaccination, i.e., just before the second injection, anti-phase II and anti-phase I IgM were present in the blood. IgM remained present for up to 90 days, after which it was no longer detectable. Regarding IgG, anti-phase II antibodies were detectable as early as 21 days after the first injection, whereas anti-phase I antibodies only appeared 21 days after the second injection. On the other hand, these latest remained for at least 9 months (the time of a booster injection), while anti-phase II IgG was at a low level from 180 days. Cellular immunity, estimated by gamma interferon (IFN-ɣ) assay, remained low until the booster dose. After the booster, IgM antibodies were once again produced in large but short-lived quantities, while IgG anti-phase II and especially anti-phase I levels were high and maintained over time. Similarly, IFN-ɣ was more significant and long-lasting. These observations are in line with experimental data showing that in mice, vaccination with a vaccine containing phase I and phase II antigens elicited both humoral and cellular immune responses [84,85]. Based on partial results of their study [81], Bauer and colleagues stated that the immune response triggered by the two different doses evaluated was equivalent. However, in the same study, IgG phase I antibodies titre was significantly higher in the 2 mL group than in the 1 mL group, 69 days after the second injection of primary vaccination. IFN-ɣ phase I titres 42 days after the second injection of primary vaccination were also significantly different depending on the dose. In an infected environment, Bauer [78,79] hypothesized that primary vaccination of previously infected ewes acted as a booster vaccination. The difference in kinetics compared with vaccination in a Q fever-free environment is probably explained by the fact that the vaccinated animals had likely already been in contact with the bacteria, and therefore, their immune systems had probably already been stimulated. However, seropositivity and infection/shedding are not well correlated [70] and serological analyses have highly variable sensitivity, depending on the test used [83]. Consequently, this hypothesis should be interpreted with caution.

#### 3.3.4. Safety and Side Effects

In an experimental study [81], a slight transient hyperthermia was noted during the first injection or the booster injection, and a slight transient skin thickening appeared during the two primary vaccination injections; however, the authors concluded that the side effects of vaccination are minor although these differences were significant between vaccinated and control group after the first injection. In another experimental study [71], no local reactions were noted after the two primary injections, but there was a slight difference in rectal temperature (+0.68 °C) compared with the control group after the first injection (*p* < 0.001). After the second injection, no significant difference was noted between the groups regarding rectal temperature. In some other studies, although many ewes were pregnant, no adverse effects from vaccination were noted [70,78].

According to the European Database of suspected adverse drug reaction report, 176 cases of adverse effects of Coxevac^®^ have been notified in sheep in Europe since 2008. In some cases, several disorders have been declared, the most common being systemic (*n* = 166), followed by disorders related to the reproductive system (*n* = 33), the mammary gland (*n* = 16) or the application site (*n* = 9). It is important to note that the vaccine was used in this species before it was licensed in sheep in 2023 [50].

## 4. Synthesis

### 4.1. Cattle

The only experimental study dealing with the use of the vaccine in cattle did not assess its efficacy. Five articles highlight the value of vaccination in improving fertility in an infected cattle herd. Only one study assessed the effect of vaccination on the reduction in the number of abortions, and although a reduction was found, it was not statistically significant. Six articles provide information about shedding. Vaccination reduces the number of animals likely to become shedders and the quantity of bacteria shed by previously infected animals. However, it appears that vaccination is more effective when carried out before pregnancy. In infected herds, vaccination also seems to be effective in reducing environmental contamination. Articles dealing with mathematical modelling (*n* = 3) show that vaccination is cost-effective and that, in an infected environment, it must be extended for at least 10 years to prevent disease re-emerging. Vaccinating the entire herd (cows and heifers) reduces the time needed to eradicate the disease. Four studies (including one experimental study) and one farmer survey have also provided information on the safety of the vaccine. Few side-effects have been noted, but there may be a temporary drop in milk production in high-producing cows that have never been vaccinated before. In addition, according to two studies, vaccination during pregnancy, although less effective, is safe.

### 4.2. Goats

Eighteen studies concern with the use of the vaccine in goats. Two experimental studies showed the vaccine’s efficacy in preventing abortions. Intervention studies also show that in an infected environment, vaccination significantly reduces the incidence of abortions. Shedding in milk, vaginal mucus and faeces was also assessed in two experimental and numerous intervention studies or case reports in infected environments. All the studies showed a reduction in the proportion of shedders or in the quantity of bacteria shed by previously infected animals. However, some goats infected before vaccination may remain shedders for several years. One study showed that vaccination reduces the risk of a goat being infected in the uterus. As in cattle, mathematical modelling shows that vaccination is cost-effective in dealing with an episode of Q fever. However, preventive use is preferable to curative use, and the vaccination protocol should be extended for 5 to 8 years. Finally, the studies that evaluated the safety of the vaccine reported a slight local reaction or a moderate rise in rectal temperature. It is also worth noting that after vaccination, *C. burnetii* DNA can be found in milk for several days, so there may be an interaction with PCR diagnosis.

### 4.3. Sheep

Fourteen studies (two experimental) deal with the vaccination of sheep, but only three followed the vaccination protocol recommended by the manufacturer. This can be explained by the fact that the authorisation for use in sheep was not granted until 2023. As a result, many earlier studies were unclear about the optimal protocol for the vaccine administration. The only experimental study evaluating vaccination to prevent abortions reported a significantly higher number of normal pregnancies in vaccinated than in unvaccinated females. More studies have assessed shedding in faeces, vaginal mucus or milk, as well as environmental contamination. One study also looked at bacteraemia. Overall, it appears that vaccination reduces the number of animals shedding the bacteria, the quantity of bacteria shed by infected animals and the environmental contamination. However, complete eradication seems difficult to achieve. Nevertheless, further studies using the manufacturer’s protocol are needed to provide reliable data. Unlike in cattle and goats, a few studies evaluating the immune response following vaccination are available in sheep. Seroconversion seems to vary between animals, but this should be interpreted with caution due to the lack of sensitivity of ELISA tests. Finally, data on vaccine safety only report mild local reaction or moderate increase in rectal temperature, even when the vaccine was used on pregnant ewes.

## 5. Conclusions

This literature review summarises the available data on the efficacy of the only commercially available vaccine against Q fever in ruminants. The different studies reviewed here show that the outcome of the vaccination with Coxevac^®^ will depend on the protocol implemented (first doses and annual booster), the timing of administration in relation to age and pregnancy, the proportion of animals vaccinated within the herd/flock, the initial seroprevalence or the level of infection. Some studies have some limitations like the lack of a control group or the use of the vaccine other than in accordance with the manufacturer’s recommendations. Similarly, the methods used to assess the outcomes of vaccination could vary from one study to another. Assessment of shedding is particularly tricky; the number of animals sampled, the type of samples collected and the method used to analyse them will have an effect on the results. Additionally, in field intervention studies, many factors cannot be controlled; for example, a natural decrease in bacterial levels or an increase in herd immunity resulting from the continuous exposure to the pathogen in heavily infected herds/flocks is not always easy to rule out. Nevertheless, although Coxevac^®^ does not completely prevent *C. burnetii* shedding, there was a general indication of the efficacy of the vaccine in reducing clinical signs and shedding of *C. burnetii* by animals in an infected herd. Reduced shedding would result in a reduction in environmental contamination, benefitting both the other animals in the herd and other cattle, sheep and goat farms in the vicinity. Finally, given the zoonotic nature of the disease, the reduction in shedding of *C. burnetii* by animals and, consequently, in environmental contamination, reduces the risk for human infection.

## Figures and Tables

**Table 1:** Distribution of articles by type of study, publication origin and species. Three articles deal with both sheep and goats.

Type of Study	Publication	Cattle	Goats	Sheep	Goats and Sheep	Total
Experimental study	Peer-reviewed journal	1	1	2		5
	Congress		1			
Intervention studies *	Peer-reviewed journal	7	7	9	3	30
	Congress	1	1	1		
	Veterinary thesis	1				
Case report	Peer-reviewed journal	1	1			5
	Congress	1	2			
Mathematical modelling	Peer-reviewed journal	2	2			5
	Congress	1				
Farmer survey	Peer-reviewed journal	1				1
Total		16	15	12	3	46

* Intervention studies include field studies and assessment of control measures.

**Table 2:** List of studies discussed in the cattle section.

Reference	Country	Year of Publication	Type of Study	Control Case Study	Statistical Analysis	Vaccination Protocol
Guatteo et al. [36]	France	2008	Intervention study	Yes	Yes	MI
Courcoul et al. [37]	France	2011	Mathematical modelling	Yes	No	NA
Ordronneau [15]	France	2012	Intervention study	Yes	Yes	MI
Taurel et al. [38]	France	2012	Intervention study	Yes	Yes	MI with annual booster
Lopez-Helguera et al. [17]	Spain	2013	Intervention study	Yes	Yes	MI
Taurel et al. [39]	France	2014	Intervention study	Yes	Yes	MI
Tutusaus et al. [40]	Spain	2014	Intervention study	Yes	No	MI
Piñero et al. [41]	Spain	2014	Intervention study	No	No	MI with annual booster before breeding
Garcia-Ispierto et al. [42]	Spain	2015	Intervention study	Yes	Yes	New vaccination given 12 months (instead of 9) after the primary vaccination
Schulze et al. [35]	Germany	2016	Experimental study	Yes	Yes	Only one injection (second injection of primary vaccination not given)
Kneblewski et al. [43]	Poland	2017	Case report	No	No	Not specified
Lehner et al. [44]	Germany	2017	Farmer survey	No	No	Not specified
Asamoah et al. [45]	China/Canada	2020	Mathematical modelling	Yes	No	NA
Yilmazbas-Mecitoglu et al. [46]	Turkey	2022	Intervention study	Yes	No	MI
Böttcher et al. [47]	Germany	2022	Case report	No	No	Once primary vaccination of animals older than 12 months. Then, primary vaccination of heifers only and single booster given in first lactation before second breeding
Raboisson et al. [48]	France	2022	Mathematical modelling	No	No	NA

MI: Manufacturer’s instructions; NA: Not applicable.

**Table 3:** Risk of being detected a medium- or a high-shedder cow associated with timing of vaccination (adapted from [38]).

	*n* Cows	*n* of Shedder Cows	Shedding Level: Medium vs. Low		Shedding Level: High vs. Low		*p* Value
			OR	IC 95%	OR	IC 95%	
No vaccination	448	76	1		1		0.03
Vaccination after AI	348	73	0.70	0.30–1.63	0.29	0.12–0.67	
Vaccination before AI	87	13	0.50	0.11–2.29	0.15	0.03–0.85	

High shedder: >10^4^ bacteria per mL of vaginal mucus; Medium shedder: [10^2^–10^4^] bacteria per mL of vaginal mucus Low shedder: [0–10^2^] bacteria per mL of vaginal mucus.

**Table 4:** List of studies discussed in the goat section.

Reference	Country	Year of Publication	Type of Study	Control Case Study	Statistical Analysis	Vaccination Protocol
Arricau-Bouvery et al. [51]	France	2005	Experimental study	Yes	Yes	MI
Rousset et al. [53]	France	2009	Intervention study	Yes	Yes	MI
Hermans et al., 2011 [54]	The Netherlands	2011	Intervention study	No	No	Not specified
Hogerwerf et al. [55] *	The Netherlands	2011	Intervention study	Yes	Yes	2 injections one month apart before pregnancy
De Crémoux et al. [56]	France	2012	Intervention study	Yes	Yes	MI
Sting et al. [57]	Germany	2013	Case report	No	No	MI
van den Brom et al. [58]	The Netherlands	2013	Intervention study	No	No	Not specified
Boarbi et al. [59]	Belgium	2014	Intervention study	No	No	Not specified
Hurtado Preciado et al. [60]	Spain	2014	Case report	No	No	MI
van Asseldonk et al. [61]	The Netherlands	2015	Mathematical modelling	Yes	No	NA
van den Brom et al. [62]	The Netherlands	2015	Intervention study	No	Yes	Not specified
Bontje et al. [63]	The Netherlands	2016	Mathematical modelling	Yes	No	NA
Gisbert et al. [64]	France/Hungary	2021	Experimental study	Yes	Yes	MI
Bauer et al. [65]	Germany	2022	Intervention study	No	No	MI on two farms. On the third farm, only young animals were vaccinated.
Jansen et al. [52]	Belgium	2022	Intervention study	Yes	No	Not specified
Bauer et al. [66]	Germany	2023	Intervention study	No	No	Not specified
Toledo et al. [67]	Spain	2023	Case report	No	No	MI

*: One study concerns both sheep and goat farms. MI: Manufacturer’s instructions. NA: Not applicable.

**Table 5:** Mean duration of shedding in vaginal mucus, faeces and milk after a *C. burnetii* challenge in vaccinated and non-vaccinated goats (adapted from [51]).

	Vaginal Shedding	Faecal Shedding	Milk Shedding
Challenged, non-vaccinated	22 days	27 days	17 days
Challenged, bivalent phase II vaccinated	16 days	28 days	14 days
Challenged Coxevac^®^ vaccinated	1.5 days	10 days	0 day

**Table 6:** Distribution of animals between groups adapted from [56].

Category	Serology Status before Vaccination	Control (C) or Vaccinated (V)	Number
Primiparous	Seropositive (non-susceptible)	C	143
		V	143
	Seronegative (susceptible)	C	113
		V	112
Multiparous	Seropositive (non-susceptible)	C	202
		V	186
	Seronegative (susceptible)	C	4
		V	2

**Table 7:** List of studies discussed in the sheep section.

Reference	Country	Year of Publication	Type of Study	Control Case Study	Statistical Analysis	Vaccination Protocol
Astobiza et al. [72]	Spain	2011	Intervention study	Yes	Yes	Two injections 3 weeks apart; dose not specified
Astobiza et al. [73]	Spain	2011	Intervention study	Yes	No	MI ***
García-Pérez et al. [74]	Spain	2012	Intervention study	No	No	Not specified
Astobiza et al. [75]	Spain	2013	Intervention study	No	Yes	MI ***
Eibach et al. [76] *	Germany	2013	Intervention study	No	Yes	1 mL—only one injection of primary vaccination
Joulié et al. [70]	France	2017	Intervention study	Yes	No	Not specified
Barkallah et al. [77]	Tunisia	2018	Intervention study	Yes	Yes	Not specified
Bauer et al. [69] *	Germany	2020	Intervention study	No	Yes	Two injections 3 weeks apart; dose not specified
Bauer et al. [78]	Germany	2021	Intervention study	No	No	1 mL—two injections 3 weeks apart
Bauer et al. [79]	Germany	2022	Intervention study	Yes	Yes	1 mL—two injections 3 weeks apart—no revaccination
Böttcher et al. [80]	Germany	2022	Intervention study	Yes	No	2 mL—two injections 3 weeks apart—vaccination of gimmers only—no revaccination
Williams-Macdonald et al. [71]	United Kingdom	2023	Experimental study	Yes	Yes	MI ***
Bauer et al. [81]	Germany	2023	Experimental study	Yes	Yes **	Either 1 mL or 2 mL dose—two injections 3 weeks apart—revaccination 9 months later

*: Two studies concern both sheep and goats farms. **: Statistics only available for the safety part of the study. ***: These studies were carried out prior to the granting of marketing authorisation for sheep, but the protocol used is identical to that of the marketing authorisation. MI: Manufacturer’s instructions.
